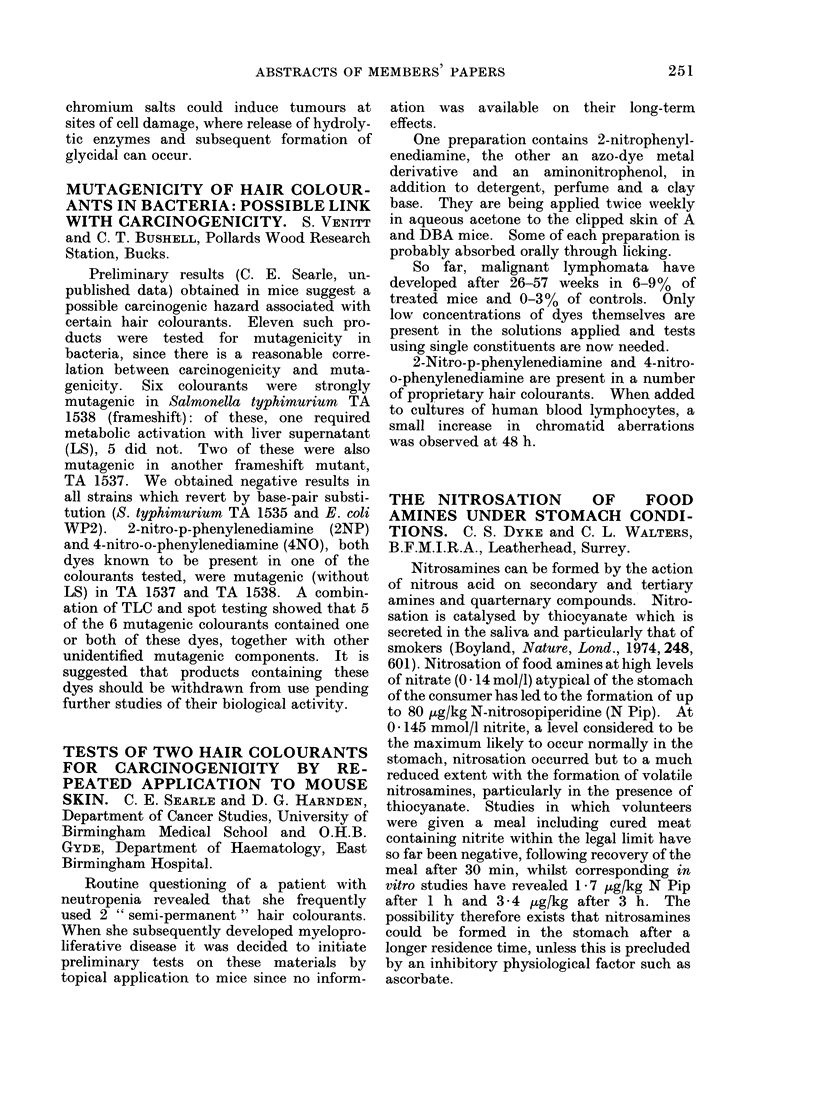# Proceedings: The nitrosation of food amines under stomach conditions.

**DOI:** 10.1038/bjc.1975.193

**Published:** 1975-08

**Authors:** C. S. Dyke, C. L. Walters


### THE NITROSATION OF FOOD AMINES UNDER STOMACH CONDITIONS.

C. S. DYKE and C. L. WALTERS, B.F.M.I.R.A., Leatherhead, Surrey.

Nitrosamines can be formed by the action of nitrous acid on secondary and tertiary amines and quarternary compounds. Nitrosation is catalysed by thiocyanate which is secreted in the saliva and particularly that of smokers (Boyland, *Nature, Lond.*, 1974, **248**, 601). Nitrosation of food amines at high levels of nitrate (0·14 mol/l) atypical of the stomach of the consumer has led to the formation of up to 80 μg/kg N-nitrosopiperidine (N Pip). At 0·145 mmol/l nitrite, a level considered to be the maximum likely to occur normally in the stomach, nitrosation occurred but to a much reduced extent with the formation of volatile nitrosamines, particularly in the presence of thiocyanate. Studies in which volunteers were given a meal including cured meat containing nitrite within the legal limit have so far been negative, following recovery of the meal after 30 min, whilst corresponding *in vitro* studies have revealed 1·7 μg/kg N Pip after 1 h and 3·4 μg/kg after 3 h. The possibility therefore exists that nitrosamines could be formed in the stomach after a longer residence time, unless this is precluded by an inhibitory physiological factor such as ascorbate.